# A Comparative Study on Aesthetic and Pain Outcomes in Flap Versus Implant Breast Reconstruction After Mastectomy

**DOI:** 10.7759/cureus.63772

**Published:** 2024-07-03

**Authors:** Kenneth Aleman Paredes, Jennifer V Castillo, Mauricio Montelongo Quevedo, Arantza Ocejo, Héctor A Vázquez Lechuga, Karime M Navarro Camara, Daniela Ponce Figueroa, Diana K Falcón García, Carina L Nolasco Mendoza, Jaqueline L Castillo, Jose A Victoria Enriquez, Jose R Flores Valdés

**Affiliations:** 1 Surgery, Hospital General Regional No. 220 "José Vicente Villada", Toluca, MEX; 2 General Medicine, Universidad Autónoma de Guadalajara, Guadalajara, MEX; 3 Gynecology, Universidad Autónoma de Guadalajara, Guadalajara, MEX; 4 Medicine and Nutrition, Universidad Juárez del Estado de Durango, Durango, MEX; 5 Surgery, Universidad Autónoma de Guadalajara, Guadalajara, MEX; 6 General Medicine, Instituto de Estudios Superiores de Tamaulipas, Tampico, MEX; 7 Health Sciences, Universidad Juárez Autónoma de Tabasco, Villahermosa, MEX; 8 General Surgery, Universidad Autónoma del Estado de México, Toluca, MEX; 9 Research, Oncology Consultants PA, Houston, USA

**Keywords:** primary breast malignancy, breast flap, plastic and reconstructive surgery, breast implant, radical mastectomy

## Abstract

Breast cancer is the most commonly diagnosed cancer and the leading cause of cancer death among women worldwide. Surgical treatments, including mastectomy and subsequent breast reconstruction, are critical components of breast cancer management. This systematic review compares the outcomes of flap versus implant reconstruction post-mastectomy, focusing on aesthetic differences, pain, recovery, and psychological adaptation.

Adhering to the Preferred Reporting Items for Systematic Reviews and Meta-Analyses (PRISMA) 2020 guidelines, we conducted a comprehensive literature search across PubMed, Cochrane, and ScienceDirect databases. Inclusion criteria targeted studies comparing aesthetic outcomes, pain, recovery costs, duration, and psychological adaptation between flap and implant breast reconstructions. We excluded non-English and non-Spanish studies, case reports, and those without full-text availability. The risk of bias was assessed using the Newcastle-Ottawa Scale (NOS). From an initial pool of 25,881 articles, 16 high-quality studies involving 14,196 participants were selected for synthesis.

Flap reconstruction was associated with higher patient satisfaction regarding aesthetic outcomes and psychological well-being but also had higher complication rates, including infections and wound dehiscence. Implant reconstruction showed fewer complications but did not achieve the same level of patient satisfaction. Flap reconstruction, despite its higher complication rates, tends to provide superior aesthetic and psychological outcomes compared to implant reconstruction. These findings highlight the importance of personalized treatment plans considering individual patient needs and preferences. Future research should focus on long-term randomized controlled trials (RCTs) and standardized outcome measures to further delineate the comparative effectiveness of these reconstruction techniques. Personalized care and ongoing research are essential to improving the quality of life for breast cancer survivors undergoing reconstruction.

## Introduction and background

The breast is a fundamental part of the human body, mainly of a woman, which allows them to nourish and connect with their children in addition to being a physically and aesthetically relevant part of their body [1]. Breast cancer is a heterogeneous disease with wide variation in tumor morphology, molecular characteristics, and clinical response. It is the most commonly diagnosed cancer type and the leading cause of cancer death in women worldwide. Invasive ductal carcinoma is the most common type of breast cancer (70%), and about 15%-20% of tumors are invasive lobular carcinomas [2]. In 2022, there were 2.3 million women diagnosed with breast cancer and 670,000 deaths globally [3]. In the United States, through the implementation of breast cancer screening and advances in treatment, a >41% mortality reduction since 1990 has been recorded [4]. There are different treatments for breast cancer, for which it is important to know the patient's stratification to offer the most beneficial treatment. However, regarding surgical treatments, mastectomy is the oldest performed surgery. There is no reliable data on the origin of mastectomy, but it is known that it has been practiced routinely in breast cancer patients since the days of the Byzantine Empire. The very first documented intervention was performed in 1882 by William Halsted, which ended up being performed on more than 90% of patients with breast cancer in the United States until the 1970s. The extent of resection led to an important associated morbidity, which inspired a lot of surgeons to modify the operation, trying to find out the best procedure [5].

Although radical mastectomy continues to be a commonly used treatment, either radical mastectomy or partial mastectomy in breast-conserving surgery causes loss of the breast and changes in appearance, leading to significant trauma and physiological impact in women [6]. Mastectomy with concurrent reconstruction can be associated with better physical well-being, improved sexual well-being, and higher health-related quality of life [7]. Female gender is the strongest breast cancer risk factor [3]. Certain factors increase the risk of breast cancer, including increasing age, obesity, the presence of certain kinds of benign tumors, increased hormone production, and family history of breast cancer. Some of the most frequent gene mutations that predispose an increased risk of breast cancer include ataxia-telangiectasia mutated (*ATM*), BRCA1-associated RING domain 1 (*BARD1*), BReast CAncer gene 1 (*BRCA1*), BReast CAncer gene 2 (*BRCA2*), cadherin 1 (*CDH1*), checkpoint kinase 2 (*CHEK2*), neurofibromatosis type 1 (*NF1*), partner and localizer of BRCA2 (*PALB2*), phosphatase and tensin homolog deleted on chromosome 10 (*PTEN*), serine/threonine kinase 11 (*STK11*), and tumor protein 53 (*TP53*) [8,9]; however, the most common high-penetrance susceptibility alleles remain *BRCA1* and *BRCA2* [2].

The importance of this topic lies in the fact that breast cancer is one of the most common cancers in the world [3], which millions of women around the world will face throughout their lives, so it is relevant to know the aesthetic options that can be offered to them, considering the differences between every patient, and be conscious of the best procedure for each of them, considering the benefits and complications that this might entail.

## Review

Methods

This study adhered to the Preferred Reporting Items for Systematic Reviews and Meta-Analyses (PRISMA) 2020 and evidence-based medicine guidelines to ensure a comprehensive systematic approach to our review [10,11].

Search Methods

Stringent inclusion and exclusion criteria were established to ensure the inclusion of only high-quality studies. The exclusion criteria were rigorously applied to maintain the quality and relevance of the studies analyzed. Studies that did not focus on aesthetic differences between flap and implant breast reconstruction, pain, recovery costs and duration, and psychological adaptation were excluded. Additionally, studies that were not available in full text or could not be obtained via interlibrary loans were excluded.

The literature search was conducted across multiple databases: PubMed (Table 1), Cochrane (Table 2), and ScienceDirect (Table 3). The search strategy employed Medical Subject Headings (MeSH) terms and free-text terms relevant to our research question. The article selection process was guided by a PRISMA flowchart. This meticulous approach enabled the creation of a homogeneous dataset, facilitating a more accurate and reliable analysis of the results.

**Table 1:** PubMed

Search strategy	Results
("breast neoplasms"[MeSH Terms] OR "breast neoplasms"[MeSH Terms] OR ("carcinoma, ductal, breast"[MeSH Terms] OR ("carcinoma"[All Fields] AND "ductal"[All Fields] AND "breast"[All Fields]) OR "breast ductal carcinoma"[All Fields] OR ("invasive"[All Fields] AND "ductal"[All Fields] AND "carcinoma"[All Fields]) OR "invasive ductal carcinoma"[All Fields]) OR ("breast neoplasms"[MeSH Terms] OR ("breast"[All Fields] AND "neoplasms"[All Fields]) OR "breast neoplasms"[All Fields] OR ("human"[All Fields] AND "mammary"[All Fields] AND "neoplasms"[All Fields]) OR "human mammary neoplasms"[All Fields]) OR ("breast neoplasms"[MeSH Terms] OR ("breast"[All Fields] AND "neoplasms"[All Fields]) OR "breast neoplasms"[All Fields] OR ("breast"[All Fields] AND "carcinoma"[All Fields]) OR "breast carcinoma"[All Fields]) OR ("breast neoplasms"[MeSH Terms] OR ("breast"[All Fields] AND "neoplasms"[All Fields]) OR "breast neoplasms"[All Fields] OR ("malignant"[All Fields] AND "neoplasm"[All Fields] AND "breast"[All Fields]) OR "malignant neoplasm of breast"[All Fields])) AND ("flap s"[All Fields] OR "surgical flaps"[MeSH Terms] OR ("surgical"[All Fields] AND "flaps"[All Fields]) OR "surgical flaps"[All Fields] OR "flaps"[All Fields] OR ("surgical flaps"[MeSH Terms] OR ("surgical"[All Fields] AND "flaps"[All Fields]) OR "surgical flaps"[All Fields] OR ("surgical"[All Fields] AND "flap"[All Fields]) OR "surgical flap"[All Fields]) OR (("pedicle"[All Fields] OR "pedicle s"[All Fields] OR "pedicled"[All Fields] OR "pedicles"[All Fields]) AND ("surgical flaps"[MeSH Terms] OR ("surgical"[All Fields] AND "flaps"[All Fields]) OR "surgical flaps"[All Fields] OR "flap"[All Fields])) OR ("deep"[All Fields] AND ("inferior"[All Fields] OR "inferiors"[All Fields]) AND ("epigastric"[All Fields] OR "epigastrical"[All Fields]) AND ("perforator flap"[MeSH Terms] OR ("perforator"[All Fields] AND "flap"[All Fields]) OR "perforator flap"[All Fields]))) AND ("breast implantation"[MeSH Terms] OR ("breast implants"[MeSH Terms] OR ("breast"[All Fields] AND "implants"[All Fields]) OR "breast implants"[All Fields] OR ("internal"[All Fields] AND "breast"[All Fields] AND "prostheses"[All Fields]) OR "internal breast prostheses"[All Fields]) OR ("breast implants"[MeSH Terms] OR ("breast"[All Fields] AND "implants"[All Fields]) OR "breast implants"[All Fields] OR ("breast"[All Fields] AND "implant"[All Fields]) OR "breast implant"[All Fields] OR "breast implantation"[MeSH Terms] OR ("breast"[All Fields] AND "implantation"[All Fields]) OR "breast implantation"[All Fields]) OR (("internal"[All Fields] OR "internally"[All Fields] OR "internals"[All Fields]) AND ("breast"[MeSH Terms] OR "breast"[All Fields] OR "breasts"[All Fields] OR "breast s"[All Fields])) OR (("breast"[MeSH Terms] OR "breast"[All Fields] OR "breasts"[All Fields] OR "breast s"[All Fields]) AND "implan*"[All Fields]) OR "breast implant"[Title/Abstract]) AND ("psychology"[MeSH Terms] OR "psychology"[All Fields] OR ("psychosocial"[All Fields] AND "Factor"[All Fields]) OR "psychosocial factor"[All Fields] OR "adaptation, psychological"[MeSH Terms] OR "psychology"[MeSH Terms] OR "psychological factor"[Title/Abstract]) AND ("randomized controlled trial"[Publication Type] OR "controlled clinical trial"[Publication Type] OR "clinical trials as topic"[MeSH Terms] OR "random allocation"[MeSH Terms] OR "double-blind method"[MeSH Terms] OR "single-blind method"[MeSH Terms] OR "clinical trial"[Publication Type] OR "research design"[MeSH Terms:noexp] OR "comparative study"[Publication Type] OR "evaluation studies"[Publication Type] OR "follow-up studies"[MeSH Terms] OR "prospective studies"[MeSH Terms] OR "cross-over studies"[MeSH Terms] OR "clinical trial"[Text Word] OR (("singl*"[Text Word] OR "doubl*"[Text Word] OR "trebl*"[Text Word]) AND ("mask*"[Text Word] OR "blind*"[Text Word])) OR "placebo*"[Text Word] OR "random*"[Text Word] OR "control"[Text Word] OR "controls"[Text Word] OR "prospectiv*"[Text Word] OR "volunteer*"[Text Word] OR ("cohort studies"[MeSH Terms] OR "case-control studies"[MeSH Terms] OR "comparative study"[Publication Type] OR "risk factors"[MeSH Terms] OR "cohort"[Text Word] OR "compared"[Text Word] OR "groups"[Text Word] OR "case control"[Text Word] OR "multivariate"[Text Word]))	54

**Table 2:** Cochrane

Search strategy	Results
("breast neoplasms" OR "breast cancer" OR "ductal carcinoma" OR "invasive ductal carcinoma" OR "mammary neoplasms" OR "breast carcinoma" OR "malignant neoplasm of breast")	46395
("flap reconstruction" OR "surgical flaps" OR "pedicle flap" OR "pedicled flaps" OR "deep inferior epigastric perforator flap" OR "DIEP")	2168
("breast implantation" OR "breast implants" OR "internal breast prostheses" OR "breast implant")	236
("aesthetic outcomes" OR "aesthetics" OR "pain" OR "psychological effects" OR "psychosocial factors" OR "adaptation, psychological" OR "psychological factor")	266452
("randomized controlled trial" OR "controlled clinical trial" OR "clinical trials as topic" OR "random allocation" OR "double-blind method" OR "single-blind method" OR "clinical trial" OR "research design" OR "comparative study" OR "evaluation studies" OR "follow-up studies" OR "prospective studies" OR "cross-over studies" OR "cohort studies" OR "case-control studies" OR "risk factors")	1153079
(#1) AND (#2 OR #3) AND (#4) AND (#5)	69

**Table 3:** ScienceDirect

Search strategy	Results
breast cancer AND flap reconstruction AND breast implants AND aesthetic outcomes AND pain AND Psychological factors AND mastectomy NOT systematic reviews NOT meta-analysis	103

Selection Criteria

Types of participants: This study has set specific participant selection criteria, including women 18 years and older who had a diagnosis of breast cancer; we excluded men, participants <18 years, and women without a diagnosis of breast cancer.

Types of intervention: Participants were included if they were treated with any type of mastectomy and who went through flap or implant breast reconstruction after mastectomy; we excluded women with breast cancer who did not go through breast reconstruction after mastectomy, women who went through more than one intervention for breast reconstruction after mastectomy, and women who underwent mastectomy for breast cancer prophylaxis.

Types of studies: We systematically reviewed relevant studies published from 2000 to 2023 in English and Spanish; we meticulously screened and analyzed randomized controlled trials (RCTs), cohort studies, and case-control trials. We excluded case reports, book chapters, case controls that did not evaluate any of the outcomes, case series, cross-sectional studies, dissertations, book chapters, protocol articles, reviews, news articles, conference abstracts, letters to the editor, editorials, and comment publications. Furthermore, we excluded studies that did not clearly describe their operationalizations, were duplicated, and could not obtain the necessary data or receive a response from the original author via email.

Type of outcomes: Primary outcomes of interest include aesthetic differences between flap and implant breast reconstruction, pain, recovery costs, duration, and psychological adaptation. These outcomes were chosen to comprehensively assess the effects of breast reconstruction type on physical appearance, patient well-being, and psychological health.

Data Extraction and Selection of Studies

During the initial phase, titles and abstracts of studies were screened by two independent reviewers (AROC and DPF) using the predetermined inclusion and exclusion criteria. Rayyan software (Rayyan Systems Inc., Cambridge, MA) [12] was used to facilitate the extraction of relevant data and filter duplicates. Keywords highlighting terms related to the inclusion and exclusion criteria were utilized in Rayyan. Any disagreements regarding study inclusion were resolved through consensus and consultation with a third reviewer (JAVE).

Following this, a detailed full-text analysis was performed, where two other reviewers (HAVL and DKFG) independently selected trials based on the same inclusion and exclusion criteria. Disagreements in this stage were similarly resolved through consensus and with the assistance of the third review author (JLC).

Data Evaluation: Assessment of Risk of Bias

Our evaluation followed the criteria outlined in the Cochrane Handbook. The Newcastle-Ottawa Scale (NOS) [13] was used for case-control and cohort studies. Two independent reviewers assessed the risk of bias in each study, considering the specific criteria and guidelines of the respective tools. Discrepancies between reviewers were resolved through discussion with a third, blinded reviewer (JLC). According to the Cochrane Handbook for Systematic Reviews of Interventions and NOS guidelines, the methodological aspects of the cohort and case-control studies were categorized as having a low, high, or unclear risk of bias. Details regarding any changes in the quality of evidence, either downgrading or upgrading, were transparently presented in the summary of findings table, along with explanations for each bias assessment.

Results

We conducted a systematic literature review to assess and compare the advantages of breast reconstruction following mastectomy using various techniques, namely, flap versus implant. Specifically, we focused on pain-related outcomes, aesthetic differences, psychological adaptation, and psychological impact. Our search, covering the period from January 2000 to the present, encompassed databases such as PubMed, Cochrane Library, and ScienceDirect; we utilized a combination of keywords, including "breast neoplasms," "surgical flaps," "breast implants," "adaptations, psychological," "aesthetic outcomes," and "pain."

A systematic review's cornerstone is methodically identifying and selecting pertinent studies from an extensive pool of literature. Our search strategy began with an expansive database query yielding 25,881 articles. Rigorous de-duplication efforts decreased this number to 16,427, with 9,454 articles excluded due to redundancy. Following a structured screening of titles and abstracts, 32 publications were earmarked for full-text evaluation. This exercise distilled the pool to 16 high-quality studies that met our strict inclusion criteria for synthesis.

**Figure 1:**
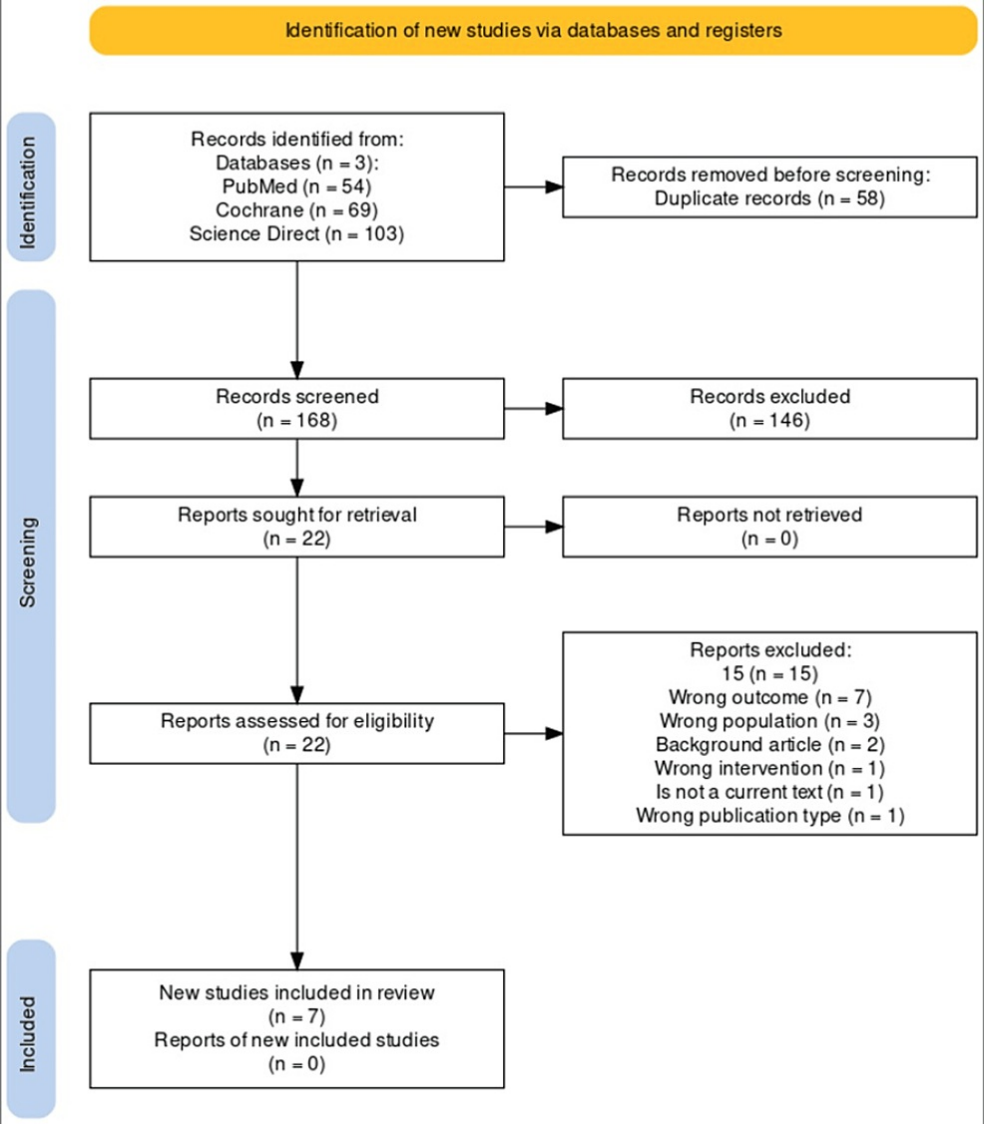
PRISMA PRISMA: Preferred Reporting Items for Systematic Reviews and Meta-Analyses

Figure 1 succinctly visualizes the study selection methodology, following the design of the PRISMA [11] flow diagram. This stepwise illustration provides transparency in our filtering process, which guided the final study selection.

This flowchart delineates the stages of literature screening for a systematic review according to PRISMA [11], mapping out the identification and inclusion of relevant studies. From three databases (PubMed (n = 54), Cochrane (n = 69), and ScienceDirect (n = 103)), a total of 226 records were identified. Before screening, 58 duplicate records were removed, yielding 168 records for screening. Subsequently, 146 records were excluded, and 22 reports were sought for detailed evaluation. All 22 reports were retrieved and assessed for eligibility. Of these, 15 were excluded for various reasons, including inappropriate outcomes (n = 7), populations (n = 3), intervention types (n = 1), and other criteria such as being a background article (n = 2), not a current text (n = 1), or the wrong publication type (n = 1). This resulted in seven studies meeting the inclusion criteria and being incorporated into the review, with their full reports being included for in-depth analysis. The flowchart effectively illustrates the rigorous study selection process to ensure the systematic review's comprehensive and relevant compilation of evidence.

Due to the main findings, the risk of bias was only assessed for cohort studies, appraising them with the Newcastle-Ottawa Scale (NOS) [13]. Our selection showed that all of the cohort studies were of good quality. The results of this assessment are presented in Table 4, providing a clear overview of the quality levels assigned to each study based on the NOS criteria.

**Table 4:** Newcastle-Ottawa Scale The table illustrates that each study received 3 stars in the selection domain, 1 or 2 stars in the comparability domain, and 2 or 3 stars in the outcome/exposure domain, qualifying them as "good quality" according to the NOS criteria. This consistent rating indicates a robust methodology and reliable findings across the assessed cohort studies. NOS: Newcastle-Ottawa Scale

Author and year	Selection domain stars	Comparability domain stars	Outcome/exposure domain stars	Quality rating
Alderman et al. (2007) [14]	3	2	2	Good
Losken et al. (2010) [15]	3	1	3	Good
Gopie et al. (2011) [16]	3	2	3	Good
Kulkarni et al. (2016) [17]	3	1	2	Good
Pusic et al. (2017) [18]	3	2	2	Good
Browne et al. (2017) [19]	3	2	2	Good
Taylor et al. (2018) [20]	3	2	3	Good

We scrutinized the impact of flap and implant-based breast reconstruction surgeries on post-surgical outcomes, including complication rates and patient satisfaction. Our systematic review encompasses a range of demographic and clinical scenarios, thus providing a robust assessment of the global experience with these surgical modalities.

In this comprehensive analysis, seven studies conducted across different geographical regions are synthesized to evaluate postoperative complications, patient-reported outcomes, and psychological impacts associated with flap and implant breast reconstruction. The point of this review covers a sample size spectrum from 83 to 6,405 participants, with an age range median hovering around the late 40s to early 50s. The collected data suggest that flap surgeries, while associated with a higher satisfaction rate in certain aspects such as self-fulfillment and sexual satisfaction, also carry a significant risk of complications. Implant surgeries, although posing a lower risk of certain complications, are not without their drawbacks, as indicated by the varying rates of major complications across studies.

A recurrent theme across several studies, particularly highlighted by Alderman et al. (2007) [14] and Pusic et al. (2017) [18], is the need for longer follow-up periods to fully understand the long-term satisfaction and quality of life of patients undergoing these reconstructive surgeries. Similarly, the interplay between psychological well-being and surgical choice is underscored by studies like those by Gopie et al. (2011) [16] and Kulkarni et al. (2016) [17], where the psychological sequelae of surgical complications are evident.

Each study contributes a piece to the puzzle, from the high complication rates noted in flap reconstructions to the nuanced satisfaction profiles associated with each reconstructive technique. Complications varied from major infections and wound dehiscence to hematoma and skin necrosis, with some studies reporting specific complication rates. For instance, Losken et al. (2010) [15] reported a 34% rate of major complications in flap surgeries, whereas Taylor et al. (2018) [20] provided a broader analysis encompassing both autologous and implant-based reconstructions.

This systematic review's quantitative synthesis can be seen in Table 5, which presents a meticulously constructed dataset of study designs, sample sizes, age demographics, intervention details, follow-up periods, complication rates, and salient points of each study. This table serves as a comprehensive reference for clinicians and researchers alike, aiming to inform practice and guide future research trajectories in the domain of breast reconstruction surgery.

**Table 5:** General outcomes SD: standard deviation

Author and year	Study design	Sample size total (number)	Age (mean ± SD)	Patients with flap intervention	Patients with implant	Post-surgical follow-up period	Surgical complications	Key points
Alderman et al. (2007) [14]	Cohort	460	N/A	292	93	2 years	N/A	This study needs to have a longer follow-up period, but in general, the satisfaction period over time takes longer time to complete.
Losken et al. (2010) [15]	Cohort	83	52.5 ± 19.5	71 (85.6%)	12 (14.4%)	26 months	34% (28/83): major infection (7.2%), minor infection (9.7%), skin necrosis (9.7%), seroma (3.6%), extrusion (1.2%)	Patients undergoing flap surgery required additional surgery unlike patients with implants, and patients with a history of radiotherapy presented major complications or more procedures.
Gopie et al. (2011) [16]	Cohort	152	Nonspecific	81 (53.28%)	71 (46.72%)	N/A	Implant (66%): major complications (13.6%), wound dehiscence (9.1%), wound infection (15.2%), bleeding (6.1%), hematoma (1.5%), skin necrosis (3%), seroma (4.5%), spitting of resorbable stitches (1.5%); flap (79%): major complications (13.9%), wound dehiscence (20.3%), wound infection (17.7%), bleeding (2.5%), hematoma (3.8%), fat necrosis (2.5%), skin necrosis (5.1%), seroma (3.8%)	This article highlights the psychological impact of post-surgical complications; the group of patients subjected to the flap surgery presented major post-surgical complications.
Kulkarni et al. (2016) [17]	Cohort	2,667	49.7 ± 10.1	453 (17%)	1,840 (69%)	N/A	N/A	Patients who share common experiences such as depression, anxiety, and breast cancer. Despite their pain, they chose to undergo reconstruction.
Pusic et al. (2017) [18]	Cohort	2,308	49.9 ± 9.9	493	1,139	12 months	N/A	Focused more on the well-being of every patient such as sexual satisfaction, self-fulfillment, and reduced levels of depression and anxiety. Patients were more satisfied with autologous reconstruction than with implant reconstruction.
Browne et al. (2017) [19]	Cohort	6,405	55.9 ± 20.3	1,617 (25.25%)	1,090 (17.02%)	18 months	629 (9.8%)	This study highlights the importance of considering surgical complications of patients with flap, mastectomy, and implant surgery when evaluating the impact of breast cancer surgery on patients' quality of life.
Taylor et al. (2018) [20]	Cohort	2,125	49.1 ± 2.7	531 (24.99%)	1,594 (75.01%)	24 months	612 (28.8%)	This study underscores the importance of considering complication rates when choosing between autologous and implant-based reconstruction, especially in unilateral versus bilateral reconstruction options.

Discussion

Our systematic review aimed to compare the outcomes of breast reconstruction techniques, specifically flap versus implant reconstruction, focusing on pain, aesthetic differences, psychological adaptation, and overall patient satisfaction. We identified seven studies that met our inclusion criteria, encompassing a total sample size of 14,196 participants, predominantly women diagnosed with breast cancer who underwent mastectomy followed by either flap or implant reconstruction. Our findings highlight that flap reconstruction generally leads to higher patient satisfaction regarding aesthetic outcomes and psychological well-being compared to implant reconstruction. However, flap reconstruction is associated with higher rates of complications, such as infections, wound dehiscence, and the need for additional surgeries.

Despite its higher complication rates, the results indicate that flap reconstruction yields better aesthetic and psychological outcomes. This suggests that the autologous tissue used in flap procedures provides a more natural look and feel, significantly contributing to patient satisfaction. The psychological benefits, including improved self-esteem and reduced anxiety and depression, underscore the importance of addressing both physical and emotional aspects of breast reconstruction. On the other hand, implant reconstruction, while associated with fewer complications, may not offer the same level of aesthetic satisfaction, which could explain the lower overall satisfaction rates observed in some studies.

The findings of this review have significant implications for clinical practice and patient care. Given that aesthetic and psychological outcomes are critical factors in the overall well-being of breast cancer survivors, it is crucial to consider these aspects when discussing reconstruction options with patients. The higher satisfaction rates associated with flap reconstruction suggest that this option should be strongly considered, especially for patients prioritizing natural appearance and long-term psychological health. However, the higher complication rates associated with flap procedures necessitate thorough preoperative counseling and postoperative care to manage potential risks effectively.

Our review also aligns with existing literature, highlighting the complexity of choosing the appropriate reconstruction method. Studies such as those by Alderman et al. (2007) [14] and Pusic et al. (2017) [18] emphasize the need for personalized treatment plans that consider individual patient needs, preferences, and medical histories. This personalized approach can help patients receive the most suitable and satisfying reconstruction option.

This review has several limitations that must be acknowledged. First, the heterogeneity of the included studies, in terms of sample sizes, follow-up periods, and specific outcomes measured, presents a challenge in drawing uniform conclusions. Second, excluding non-English and non-Spanish studies may have resulted in the omission of relevant data. Third, the reliance on observational studies, which are inherently prone to bias, limits the ability to establish causality. Additionally, the varying definitions and measurements of psychological and aesthetic outcomes across studies could affect the comparability of results. Lastly, the potential for publication bias exists, as studies with significant findings are more likely to be published.

Future research should focus on long-term, randomized controlled trials to provide more definitive evidence on the comparative effectiveness of flap versus implant reconstruction. Studies should aim to standardize outcome measures, particularly in terms of psychological and aesthetic assessments, to facilitate more robust comparisons. Additionally, exploring the role of patient-specific factors, such as genetic predispositions, comorbidities, and personal preferences, can help tailor reconstruction options more effectively. Finally, further research into developing and implementing enhanced recovery protocols and postoperative care strategies could mitigate the higher complication rates associated with flap reconstruction, thereby optimizing patient outcomes.

While both flap and implant breast reconstruction techniques have their advantages and drawbacks, the choice should be guided by a comprehensive evaluation of patient preferences, aesthetic goals, and potential risks. Personalized care and ongoing research are essential to improving the quality of life for breast cancer survivors undergoing reconstruction.

## Conclusions

This systematic review highlights the distinct advantages and disadvantages associated with flap and implant breast reconstruction techniques post-mastectomy. Flap reconstruction, despite being linked to higher rates of complications such as infections and wound dehiscence, offers superior aesthetic outcomes and significant psychological benefits, including enhanced self-esteem and reduced anxiety and depression. These findings underscore the importance of considering the holistic well-being of breast cancer survivors when making reconstruction decisions. Implant reconstruction, while associated with fewer complications, does not achieve the same level of patient satisfaction regarding aesthetic and psychological outcomes. This suggests that while implants may be a safer option in terms of immediate postoperative complications, they may not fully address the long-term psychological and aesthetic needs of patients. These findings have substantial implications for clinical practice. Personalized treatment plans that consider the patient's preferences, medical history, and aesthetic goals are essential. Preoperative counseling should include discussions about each reconstruction option's potential risks and benefits, ensuring patients make informed decisions that align with their long-term well-being.
